# Cardiac autonomic nervous activity in patients with transposition of the great arteries after arterial switch operation^☆^

**DOI:** 10.1016/j.ijcchd.2022.100417

**Published:** 2022-08-17

**Authors:** Covadonga Terol Espinosa de los Monteros, Roel L.F. van der Palen, Ineke Nederend, Eco J.C. de Geus, Irene M. Kuipers, Mark G. Hazekamp, Nico A. Blom, Arend D.J. ten Harkel

**Affiliations:** a Department of Pediatrics, Division of Pediatric Cardiology, Leiden University Medical Center, Leiden, the Netherlands; b Department of Biological Psychology, Human Behavioral and Movement Sciences, Vrije University, Amsterdam, the Netherlands; c Department of Pediatrics, Division of Pediatric Cardiology, Amsterdam UMC, University of Amsterdam, Amsterdam, the Netherlands; d Department of Pediatric Cardiac Surgery, Leiden University Medical Center, Leiden, the Netherlands

**Keywords:** Transposition of the great arteries, Arterial switch operation, Autonomic nervous system, Left ventricular function

## Abstract

**Background:**

A chronic imbalance of the autonomic nervous system(ANS) may contribute to long term complications in different congenital heart diseases. The purpose of this study was to determine whether the ANS plays a role in the long-term outcome of patients with Transposition of great arteries(TGA) after arterial switch operation(ASO) as its contribution is as yet not clear.

**Methods:**

The ANS activity was evaluated non-invasively in 26 TGA patients and 52 age-appropriate healthy subjects combining impedance cardiography and electrocardiography. Heart rate, pre-ejection period(sympathetic activity parameter) and respiratory sinus arrhythmia and the root of the mean square of successive normal-to-normal interval differences(parasympathetic activity parameter) were measured during 5 different daily activities(sleep, sitting, active sitting, light and moderate/vigorous physical activity). Whether the ANS activity was related to ventricular function, exercise test performance or clinical outcome in the patient group was also analyzed.

**Results:**

Compared to healthy subjects: heart rate was significantly lower in TGA patients at rest and during quiet and active sitting; sympathetic activity was significantly reduced in patients during physical activity; and the parasympathetic activity was higher in TGA patients while quiet and active sitting. In the patient group a significant positive correlation between 4-chamber longitudinal strain and parasympathetic activity during 3 different daily activities was found.

**Conclusions:**

The sympathetic nervous system response to physical activity is reduced in TGA patients after ASO. Additionally, we observed a positive correlation between better left ventricular function and higher parasympathetic activity that could be in line with the known protective effect of a higher vagal activity.

## Highlights

• A chronic imbalance of the autonomic nervous system may contribute to long term complications in congenital heart diseases.
• The cardiac autonomic nervous system activity could be assessed on an outpatient basis by combining impedance cardiography and electrocardiography.
• The sympathetic nervous system response to physical activity is reduced in patients with Transposition of the great arteries after arterial switch operation.
• A positive correlation between better left ventricular function and higher parasympathetic activity reinforces the idea of a protective effect of a higher vagal activity.

## 1 Introduction

The autonomic nervous system (ANS) plays an important role in the homeostasis of the cardiovascular system and its response to environmental changes, as it directly regulates heart rate [1]. In several cardiac diseases the balance between sympathetic and parasympathetic nervous activity has been disturbed which may contribute to long-term cardiovascular complications. A chronic imbalance with an increased sympathetic activity and decreased parasympathetic activity has been reported to progress heart failure [2] in patients with congenital heart diseases (CHD), and such imbalance in these patients has also been associated with an increased risk of cardiac events and even sudden cardiac death [3,4]. However, it is not known to what extent the cardiac autonomous nervous activity is impaired after corrective surgery for CHD. Therefore, a detailed knowledge of the ANS activity in these patients is of paramount importance. Transposition of the great arteries (TGA) is one of the most common cyanotic heart defects and represents 5–7% of the CHD with a prevalence of 2.3 per 10.000 live births. The arterial switch operation (ASO) has become the standard surgical repair worldwide with proven low early and late mortality rates [5,6]. Because most of the sympathetic nerves enter the heart in the cardiac plexus via the great arteries [1,7], the ASO might have a negative effect on the sympathetic innervation [8]. Long-term outcome studies show that TGA patients after ASO commonly have a diminished exercise tolerance [[9], [10], [11], [12]] and that approximately 5% of the TGA patients have severe cardiac events such as arrythmias, myocardial infarction, heart failure and even sudden cardiac death [5]. Only few studies previously studied the ANS in TGA patients after ASO, and it remains unclear whether the ANS plays a role in the long-term outcome of these patients. Therefore, the purpose of this study was to evaluate the ANS in TGA patients with a mid-term follow-up period after ASO and to determine if alterations in the ANS could be related to differences in ventricular function or exercise performance. By combining impedance cardiography (ICG) and electrocardiography (ECG) cardiac ANS activity could be assessed on an outpatient basis.

## 2 Methods

### 2.1 Study population

TGA patients after ASO were prospectively enrolled in this study between December 2015 and July 2017. Patients had either TGA with an intact ventricular septum (IVS) or with a ventricular septal defect (VSD); patients with more complex anatomy such as Taussig-Bing anomaly, aortic arch pathology and double outlet right ventricle with TGA were not included in this study. All patients underwent an ASO at the Leiden University Medical Center (LUMC) between 1997 and 2006 and had their cardiovascular follow-up at 1 of 2 collaborating academic medical centers (LUMC or Amsterdam University Medical Center). In addition, a group of healthy children with a similar age range was enrolled in the study between January 2014 and December 2015 and served as a reference group. The healthy subjects were referred to the Pediatric Cardiology department of the LUMC with an asymptomatic, innocent heart murmur, cardiac palpitations or idiopathic chest pain. Subjects were included if routine echocardiographic evaluation showed a structurally normal heart with normal cardiac function.

TGA patients and healthy subjects underwent both a 24-h ambulatory ANS monitoring and transthoracic echocardiography. Additionally, TGA patients underwent a cardiopulmonary exercise test (CPET). Echocardiography and CPET examinations from TGA patients were performed within 6 months around the ANS monitoring. For the healthy subjects, the transthoracic echocardiography was performed on the same day as the ANS monitoring. Hospital and outpatient records were reviewed to obtain demographics, preoperative anatomy and surgery details and demographics for the healthy subjects were acquired via a questionnaire.

A lifestyle interview was performed to evaluate patients’ weekly exercise behavior according to the previously described method [13]. The voluntary exercise behavior (e.g. swimming, fitness, tennis, jogging, soccer), physical activities related to transportation (cycling, walking) and compulsory physical education classes that were done for at least 6 months and more than 3 months per year were included. Each exercise activity was converted into a metabolic equivalent task (MET) score [14], and a weekly MET score (MET hours/week) was calculated (i.e. MET scores multiplied by the duration of activities and summed).

The medical records of the patients were reviewed at July 2022 (5–7 years after the study) to determine cardiac events (morbidity and mortality). Cardiac morbidity was defined as the need of cardiac-related hospitalization including medical or surgical management of any complications derived from heart failure or arrhythmias.

The study protocol conforms to the ethical guidelines of the 1975 Declaration of Helsinki as reflected in a priori approval by the institution's human research committee and written informed consent was obtained from the participants and their legal guardians where appropriate.

### 2.2 Echocardiographic and cardiopulmonary exercise test parameters

Echocardiography was performed according to a standardized clinical follow-up protocol using a commercially available ultrasound system (Vivid 7.0 and 9.0, GE Vingmed Ultrasound, AS, Horten, Norway) equipped with 3.5–7.0 MHz transducers. Images were stored in digital format to allow off-line analysis using EchoPac version 11.3 (General Electric Vingmed). All patients were in sinus rhythm with stable heart rate at the time of the echocardiographic investigation. Speckle tracking strain imaging analysis was performed in the 2-dimensional gray-scale images from the apical 4 chamber to assess longitudinal peak strain (LS) of the left ventricle (LV) following the standard recommendations [[15], [16], [17]]. Neo-aortic valve regurgitation was graded semi-quantitatively from the parasternal long axis by color Doppler imaging according to the international guidelines [18] and categorized as follows: absent, trivial, mild, moderate or severe. The presence of aortic valve stenosis was also studied according to the guideline recommendations [19].

TGA patients performed a progressive CPET on an electronically braked cycle ergometer (GE Healthcare eBike Comfort, Freiburg, Germany) up to exhaustion. A facemask (Hans Rudolph, Kansas City, MO, USA) connected to a flowmeter (Triple V volume transducer) and a computerized gas analyzer (Jaeger MasterScreen CPX, CareFusion GmbH, Hoechberg, Germany) which calculated breath-by-breath minute ventilation (VE), oxygen uptake (VO_2_), carbon dioxide production (VCO_2_) and respiratory exchange ratio (RER, defined as the ratio VCO_2_/VO_2_) in 10 s intervals were used. The analysis of the evaluated data (oxygen uptake efficiency slope (OUES); oxygen uptake at peak exercise (*VO*_*2peak*_), peak work rate (*WR*_*peak*_), peak heart rate (HR_peak_)) and reference values used was previously described by our research group [12].

### 2.3 Ambulatory cardiac ANS measurement

The cardiac ANS was measured ambulatory with the 5th version of the Vrije Universiteit Amsterdam (VU) Ambulatory Monitoring System (VU-AMS; VU University, The Netherlands) that registered the 24-h ECG and ICG. Three pregelled Ag/AgCl (Kendal H124SG, Halberstadt, Germany) spot electrodes on the chest were used to obtain a 1-lead ECG. The thoracic impedance (Z) against a small alternating current (50 kHz, 350 μA) through the thorax was measured using spot electrodes. The electrodes were placed just above and below the sternum (measuring electrodes) and in the back (current electrodes). The ICG or DZ/dt was derived by differentiating the changes in the impedance (dZ) over time, and R-wave time-locked ensemble averaging across all beats in each of the ambulatory activities. Ectopic beats were removed from ICG signal processing.

The pre-ejection period (PEP) was defined as the time interval between ventricular depolarization (Q wave on the ECG) and start of LV outflow (B point in the ICG); and it was used to measure the sympathetic nervous system (SNS). The parasympathetic nervous system (PNS) was measured with the respiratory sinus arrhythmia (RSA) using the peak valley method. The shortest interbeat-interval during inhalation and the longest during exhalation were subtracted using the respiration signal from the low-pass filtered dZ signal, and the inter-beat interval from the ECG. If no shortest or longest interbeat-interval could be detected, RSA was set to 0 for that breath. Additionally, the root of the mean square of successive normal-to-normal interval differences (RMSSD), and its correction for the mean interbeat interval (cRMSSD) [20], were calculated to determine the heart rate variability that mainly reflect the PNS activity in the heart [20,21]. A higher SNS activity is represented by a shorter PEP and a higher PNS activity is represented by a higher RSA, RMSSD and cRMSSD.

The 24-h recording was divided into fixed periods using an activity diary completed by the participants and the accelerometer data from the VU-AMS device. These periods were coded for activity in 5 groups: sleeping, sitting (watching TV, reading, computer), active sitting (class, crafts, homework), light physical activity (walking, chores), moderate to vigorous physical activity (cycling, gymnastics, playing). The mean heart rate (HR), PEP,RSA, RMSSD and cRMSSD were calculated for each patient for each of the 5 ambulatory activities.

### 2.4 Statistical analysis

SPSS Statistics software (version 25.0 IBM SPSS, Chicago, IL) was used to perform data analysis. The Kolmogorov-Smirnov or Shapiro-Wilk test were used to determine the distribution of the data. The data are provided as mean ± standard deviation (SD) or median and interquartile range [IQR] depending on the distribution for continuous variables, and percentage for discrete variables. Some CPET parameters were expressed relatively to the reference values as % of predicted value (100% would mean equal to reference value) and represented as mean with 95% of confidence interval (CI). Chi squared test was performed to assess differences in gender distribution between TGA group and healthy subject group and differences in the ANS parameters between TGA patients that had or not a cardiac event in the follow up period. A non-paired *t*-test or the Mann-Whitney test were used to analyse the differences between the 2 cohorts for age, anthropometric data, ANS measures and the speckle tracking strain parameters. Pearson's correlation or Spearman's rank test were used for the correlation between the ANS measures and the echocardiographic and CPET parameters. A p-value < 0.05 was considered statistically significant.

## 3 Results

A total of 26 TGA patients and 52 age-appropriate healthy subjects were enrolled in this study.

Baseline characteristics are depicted in Table 1. No differences were observed between patients and controls for age and anthropometry metrics. The morphological TGA subtypes were: TGA/IVS in 20 (77%) and TGA/VSD in 6 (23%) patients. Twenty-three TGA patients underwent one-stage repair ASO (88.5%) and the Lecompte maneuver was performed in all of them. None of the patients were under medication. There were no significant differences in the average weekly MET score between TGA patients and healthy subjects (healthy subjects 56.6 ± 24.5 and TGA 53.7 ± 26.3; p = 0.658).

**Table 1:** Baseline characteristics and longitudinal strain values of the left ventricle.

	TGA (n = 26)	Healthy subjects (n = 52)	p
Sex M (%)	19 (73.1%)	32 (61.5%)	0.313
Age, years	14.2 ± 2.2	14.2 ± 2.4	0.993
**Clinical characteristics of TGA patients**			
VSD	6 (23%)		
Coronary anatomy (1LCx-2R)	17 (65%)		
Age at ASO (days)	7 [5–14.5]		
Weight at ASO (kg)	3.5 ± 0.5		
**Anthropometry**			
Weight, kg	55.6 ± 15	53.9 ± 14.9	0.635
Height, cm	166.8 ± 12.9	164.5 ± 14.3	0.494
Body mass index	19.6 ± 2.7	19.5 ± 2.8	0.873
Body surface area	1.6 ± 0.3	1.6 ± 0.3	0.601
Systolic blood pressure, mmHg	126 [113–140]	114 ± 11	**0.002**
Diastolic blood pressure, mmHg	65 [54–77]	66 ± 8	0.844

Data expressed as media ±SD or median [IQR].

*ASO*: atrial switch operation; *TGA*: transposition of the great arteries; *VSD*: ventricular septal defect.

Twenty patients (83%) had no events during a 5 years follow up period and 2 were lost to follow up. Two patients needed surgery (one aortic valve replacement and one pulmonary valve replacement), 1 had an endocarditis and 1 presented with transient rhythm disorders that did not need medication.

The 4 chamber-LS of the LV was significantly diminished in TGA patients compared to healthy subjects (−15.2% ± 2.3 in TGA vs −18.7% ± 1.5 in healthy subjects, p < 0.001). Only 7.7% of the TGA patients had moderate aortic regurgitation, 61.5% mild and 30.8% absent or trivial; none of them had severe regurgitation. TGA patients showed nor aortic valve stenosis neither aortic arch obstruction. CPET results of the TGA patients are summarized in Table 2. All TGA patients exercised to exhaustion with an RER >1.01. Work rate and heart rate values were comparable to age and sex matched normal values, while reduced values of the % predicted values of both maximal and submaximal exercise parameters (VO_2peak_% 75%, O_2_ pulse_max_ 82%, OUES 88%) were observed.

**Table 2:** Exercise test results of patients with Transposition of the great arteries (TGA).

TGA (n = 26)	
RER	1.2 [1.1–1.3]
WR_peak_ (W)	177 ± 57
WR_peak_%	98 (90–105)
HR_peak_ (bpm)	187 [176–187]
HR_peak_%	99 (94–100)
VO_2peak_ (ml/min)	2082 ± 600
VO_2peak_%	75 (70–81)
OUES (ml/min/log(L/min))	2137 ± 549
OUES%	88 (80–95)
VE/VCO_2_ slope (L/min)	28.4 ± 4

Data expressed as mean±SD, median [Q1-Q3] and N(%). The %predicted values were shown as mean(95%CI).

*HR*_*peak*_: maximal heart rate at peak exercise; *HR*_*peak*_*%*: % of the predicted values of HR_peak_; *IVS*: interventricular septum; *OUES*: oxygen uptake efficiency slope; *OUES*%: % of the predicted values of OUES; *RER*_*peak*_: respiratory exchange ratio at peak exercise; *RV*: right ventricle; *VCO*_*2*_: carbon dioxide production; *VE*: minute ventilation; *VO*_*2peak*_: oxygen uptake at peak exercise; *VO*_*2peak*_*%*: % of the predicted values of VO_2peak_; *VSD*: ventricular septal defect; *WR*_*peak*_: peak work rate; *WR*_*peak*_*%*: % of the predicted values of WR_peak_.

### 3.1 Autonomic nervous system analysis

**Fig. 1:**
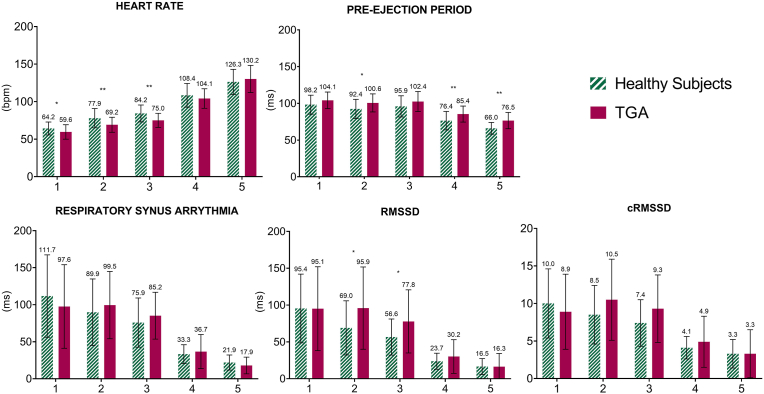
Means and standard deviation of the ANS parameters. Mean and SD (error bars) of the ambulatory measurements of heart rate, pre-ejection period, respiratory sinus arrythmia, root of the mean square of successive normal-to-normal interval differences (RMSSD) and its correction for the interbeat interval (cRMSSD) in healthy subjects and TGA patients for each of the 5 activity levels: sleeping (1), quiet sitting (2), active sitting (3), light physical activity (4), moderate to vigorous physical activity (5). *p < 0.05; **p < 0.005.

Fig. 1 and Supplemental Table S1 summarize the group means of HR, PEP,RSA, RMSSD and cRMSSD in each ambulatory activity. The HR was significantly lower in TGA patients than in healthy subjects only during sleep and quiet and active sitting but not during light or moderate/vigorous physical activity. PEP was significantly longer in TGA patients compared to healthy subjects only during physical activity. No differences in RSA were observed between groups. Only in quiet and active sitting the TGA patients showed lower values of RMSSD compared to healthy subjects but not in the cRMSSD.

**Fig. 2:**
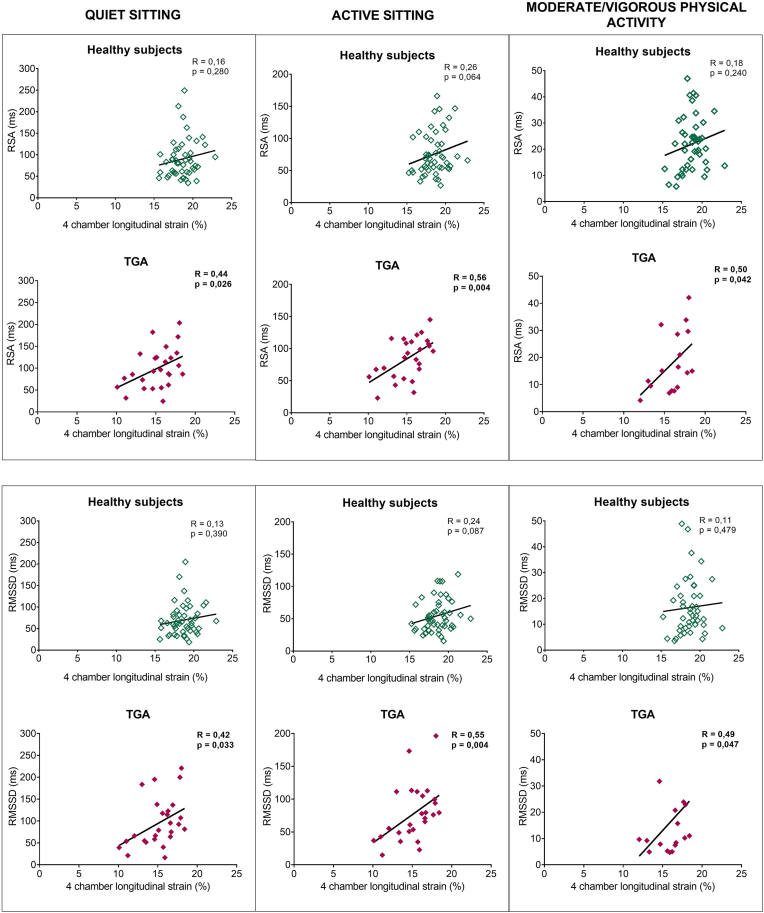
Correlation between respiratory sinus arrythmia and root of the mean square of successive normal-to-normal interval differences with the apical 4 chamber longitudinal strain in TGA patients and healthy subjects. The correlation between respiratory sinus arrythmia (RSA) and root of the mean square of successive normal-to-normal interval differences (RMSSD) in 3 different activity levels and the apical 4 chamber longitudinal strain was statistically significant in the TGA group but not in the healthy subjects group.

Neither SBP nor DBP were correlated with PEP values (Table S2). The PEP values were not different between TGA patients with or without aortic regurgitation (Table S3). The correlation analysis between the ANS parameters and the LV systolic function (4chamber LS) are depicted in Table S4. A significant correlation was found between the 4-chamber longitudinal strain and the RSA and RMSSD (Fig. 2) while sitting, active sitting and moderate/vigorous physical activity. Higher PEP values at quiet and active sitting and at moderate physical activity were correlated to higher absolute values of peak work rate, O_2_ pulse and VO_2_ values, but were, however, not correlated to the percentages of predicted (Table S5). There was no correlation between the MET score and the ANS parameters (Table S6). We did not find consistent significant differences (present in more than 2 activities) between patients with or without cardiac events in the ANS parameters.

## 4 Discussion

In the present study PNS activity was comparable between TGA patients and healthy controls. In contrast, PEP was longer in TGA patients, especially during higher activity levels, reflecting a reduced SNS activity in these patients. Reduced ventricular function was correlated to lower overall daily PNS activity. By using an ambulatory system it was possible to monitor ANS during regular daily activities.

The balance in ANS activity plays a key role in the pathophysiology of heart failure [2]; and its role has also been recognized in patients with different forms of CHD [[22], [23], [24], [25], [26]]. This imbalance is usually characterized by increased SNS activity and decreased PNS activity. Moreover, an impaired ANS activity is related to increased morbidity and mortality in different cardiovascular diseases [27,28] and it has been described to predict sudden cardiac death in patients with CHD [3]. Therefore, it is important to be informed about the actual ANS activity in individual CHD patients.

In this study, we reported that TGA patients after ASO, instead of a hyperactive SNS activity, indeed had lower SNS activity (determined by a longer PEP) than healthy controls. This contrasts to patients after surgical VSD closure who showed normal autonomic nervous activity 10 years after correction [25]. During the ASO the great vessels are dissected and this may damage the sympathetic nerves as the cardiac plexus travels to the heart through the great arteries [1,7]. Although this may result in sympathetic denervation of the heart immediately after surgery, on the long-term the heart will usually at least partly be reinnervated [8]. This is even more likely in patients operated in the neonatal period, which is usually the case for ASO [8]. Based on this study, TGA patients fifteen years after ASO had significantly lower SNS activity than controls during physical activity. Previously it has been described that patients with re-innervation of the SNS after ASO were more likely to have normal exercise capacity [8]. In the present study the TGA patients had reduced exercise capacity as compared to the general pediatric population (as reflected by low percentage predicted values of VO_2_ and OUES), which was not correlated to ANS activity.

The lower SNS activity may also be related to a diminished LV function. In patients after aortic coarctation repair, a correlation between impaired LV function and longer PEP has been described [26]. The strain values of the LV in this study reflect an LV impairment of the TGA patients after ASO, but we have not found a significant lower SNS activity.

It is known that the PEP can be influenced not only by the SNS but also by other cardiovascular variables such as pre and afterload conditions and the diastolic blood pressure [29]. There were no differences in DBP between TGA and healthy subjects and it was not correlated with the PEP. All the participants were in good clinical condition (NYHA class I) and without any medication so we assumed that they were euvolemic although no objective measurements of volume preload was performed. The afterload of the LV can be affected by several factors. The SBP was higher in TGA patients than in healthy subjects but it was not correlated to the PEP. Neither aortic valve stenosis nor arch problems were present in the study population. And we found no significant differences in the PEP values between patients with or without aortic regurgitation. Few studies have focused on the evaluation of the PNS activity in TGA patients. It seems that before surgery the PNS activity is decreased but after surgery the results are not consistent. Harrison et al. studied the ANS function in patients before and after surgery (immediately, 6 weeks and 3 years post-ASO) and found normal PNS activity during rest but lower PNS activity while doing an age-appropriate task (feeding, block stacking) compared to controls [30,31]. Conversely, the PNS activity was higher than healthy controls in another study of TGA patients 12–18 months after surgery [32]. This study included an older group of patients (14 years after ASO) and showed no significant difference between TGA patients after ASO and healthy controls with regard to PNS activity. Nevertheless, we found a significant correlation between higher RSA and RMSSD values and better LV systolic function (determined by 4 chamber longitudinal strain). This was also found by Nederend et al. in patients after surgical correction for aortic coarctation and is in line with the idea that a higher PNS activity could have a protective effect on the patients with heart diseases [27,28]. In a previous study of Tran et al. patients with diastolic LV dysfunction the PNS was stimulated by low-level transcutaneous vagal nerve stimulation [33]. This led to increased HR variability and an improvement in LV longitudinal strain. Therefore, the trend we found with the relationship of PNS activity and LV function is in line with previous studies and may give hope for possible future therapy in patients with sympathovagal imbalance.

The current study is subject to some limitations. The ANS is a very dynamic and complex system that is influenced by diverse factors. The use of a non-invasive method (ICG + ECG) to study it can potentially introduce measurement errors in our analysis and so on our results and conclusions based on them. Nevertheless, it allows us to study the ANS performance and could help us to better understand the pathophysiology of these patients on an outpatient, accessible and non-invasive basis. Besides, the ANS analysis was made in a relative small sample of young adolescent TGA patients after ASO and may therefore not be representative for TGA patients at older ages. Moreover, as the development of diminished cardiac function in TGA patients after ASO usually appears beyond childhood, a study of the ANS in an adult population could help to understand the underlying mechanism of systolic cardiac dysfunction in this group of patients and to decide treatment strategies in the long-term period. Furthermore, a CPET was not performed in the healthy subjects that participated in this study so we could not compare the results with the TGA patients. Instead we used the percentage predicted values to compared the CPET results of the TGA patients to healthy database. Finally, there are different factors, including mental health and other psychosocial factors that can influence in the vagus nerve function that were not explored in this study.

## 5 Conclusions

In the present study the response of the SNS to physical activity is reduced in TGA patients mid-term after ASO compared to healthy controls. Additionally, the positive correlation we found between better systolic LV function and higher PNS activity could be in line of the previously described protective effect on the cardiac function of a higher vagal activity.

## Disclosures

### Ethical statemen

All human and animal studies have been approved by the appropriate ethics committee and have therefore been performed in accordance with the ethical standards laid down in the 1964 Declaration of Helsinki and its later amendments.

### Funding

This research did not receive any specific grant from funding agencies in the public, commercial, or not-for-profit sectors.

### Author contributions

Conceptualization: Covadonga Terol Espinosa de los Monteros, Roel LF Van der Palen, Nico A Blom, Aren DJ ten Harkel. Methodology: Covadonga Terol Espinosa de los Monteros, Roel LF Van der Palen, Aren DJ ten Harkel. Data Curation: Covadonga Terol Espinosa de los Monteros, Roel LF Van der Palen, Ineke Nederend. Formal analysis and investigation: Covadonga Terol Espinosa de los Monteros. Writing – Original Draft: Covadonga Terol Espinosa de los Monteros. Writing- Reviewing and Editing: Roel LF Van der Palen, Ineke Nederend, Eco J.C. de Geus, Irene M. Kuipers, Mark G: Hazekamp, Nico A Blom, Aren DJ ten Harkel. Validation: Covadonga Terol Espinosa de los Monteros, Roel LF Van der Palen, Ineke Nederend, Eco J.C. de Geus, Irene M. Kuipers, Mark G: Hazekamp, Nico A Blom, Aren DJ ten Harkel.

## Declaration of competing interest

The authors declare that they have no known competing financial interests or personal relationships that could have appeared to influence the work reported in this paper.
