# Supplementary material for: Cardiac autonomic nervous activity in patients with transposition of the great arteries after arterial switch operation
Source: Int J Cardiol Congenit Heart Dis. 2022 Aug 17;10:100417. doi: 10.1016/j.ijcchd.2022.100417 (PMC11658539; doi:10.1016/j.ijcchd.2022.100417)
Supplement: Multimedia component 1 [file mmc1.docx]

**Supplementary Table S1. Autonomic system parameters in TGA patients and healthy subjects.**

|  | **Activity level** | **TGA patients (n=26)** | **Healthy subjects (n=52)** | **p** |
| --- | --- | --- | --- | --- |
| **HR, beats/min** | Sleep | 59.6 ± 9.6 | 64.3 ± 8.6 | **0.035** |
|  | Quiet sitting | 69.2 ± 10 | 77.9 ± 12.8 | **0.004** |
|  | Active sitting | 75 ± 9.4 | 84.2 ± 11.3 | **0.001** |
|  | Moderate PA | 104.1 ± 13 | 108.4 ± 15.8 | 0.239 |
|  | Heavy PA | 130.2 ± 18 | 126.3 ± 16.7 | 0.432 |
| **PEP, ms** | Sleep | 99.2 ± 11.3 | 98.2 ± 12.9 | 0.064 |
|  | Quiet sitting | 100.6 ± 12.3 | 92.4 ± 13 | **0.011** |
|  | Active sitting | 102.4 ± 13.6 | 95.9 ± 14.4 | 0.064 |
|  | Moderate PA | 85.4 ± 10.9 | 74.5 [66.1-87.5] | **0.001** |
|  | Heavy PA | 76.5 ± 11.1 | 66 ± 7.9 | **<0.001** |
| **RSA, ms** | Sleep | 82.8 [64.7-111.9] | 97.4 [76.8-136.1] | 0.202 |
|  | Quiet sitting | 99.5 ± 45.4 | 80.7 [58.7-108.1] | 0.275 |
|  | Active sitting | 85.2 ± 31.6 | 68.7 [52.5-97.2] | 0.149 |
|  | Moderate PA | 31.2 [17.6-44.9] | 31.2 [22.2-42.5] | 0.653 |
|  | Heavy PA | 17.9 ± 11.3 | 21.9 ± 10.2 | 0.187 |
| **RMSSD, ms** | Sleep | 95.1 ± 56.9 | 95.5 ± 46.6 | 0.976 |
|  | Quiet sitting | 95.9 ± 55.8 | 62.2 [42.4-83.7] | **0.033** |
|  | Active sitting | 73.4 [47.4-106.5] | 49.6 [37.3-70.8] | **0.013** |
|  | Moderate PA | 22.4 [14.9-40.6] | 21.2 [14.3-40.1] | 0.518 |
|  | Heavy PA | 9.7 [6.4-21.8] | 13.3 [9.7-21] | 0.267 |
| **cRMSSD** | Sleep | 8.9 ± 5 | 9.9 ± 4.6 | 0.387 |
|  | Quiet sitting | 10.5 ± 5.4 | 7.9 [5.7-9.8] | 0.102 |
|  | Active sitting | 9.3 ± 4.5 | 6.6 [5.2-8.7] | 0.058 |
|  | Moderate PA | 4 [2.9-5.7] | 3.7 [2.9-5.1] | 0.653 |
|  | Heavy PA | 2 [1.5-4] | 2.8 [2-4.3] | 0.274 |

Data expressed as media ± SD or median [IQR]

*HR*: heart rate; *PEP*: pre-ejection period; *RMSSD*: root of the mean square of successive normal-to-normal interval differences; *cRMSSD*: root of the mean square of successive normal-to-normal interval differences corrected by interbeat interval; *RSA*: respiratory sinus arrythmia

**Supplementary Table S2. Correlation analysis between blood pressure and pre-ejection period.**

|  | **Activity level** | **TGA patients (n=26)** | **Healthy subjects (n=52)** |
| --- | --- | --- | --- |
| **PEP vs SBP** | Sleep | R=0.139; p=0.527 | R=0.150; p=0.295 |
|  | Quiet sitting | R=0.242; p=0.244 | **R=0.287; p=0.048** |
|  | Active sitting | R=0.125; p=0.550 | R=-0.121; p=0.399 |
|  | Moderate PA | R=0.363; p=0.075 | R=0.208; p=0.147 |
|  | Heavy PA | R=0.306; p=0.251 | R=0.033; p=0.833 |
| **PEP vs DBP** | Sleep | R=-0.520; p=0.812 | R=-0.102; p=0.477 |
|  | Quiet sitting | R=0.177; p=0.397 | R=0.200; p=0.173 |
|  | Active sitting | R=0.153; p=0466 | R=-0.34; p=0.811 |
|  | Moderate PA | R=0.198; p=0.343 | R=0.039; p=0.787 |
|  | Heavy PA | R=0.069; p=0.799 | R=-0.081; p=0.602 |

Pearson or Spearman correlation when appropriate.

*DBP*: diastolic blood pressure; *PEP*: pre-ejection period; *SBP*: systolic blood pressure.

**Supplementary Table S3. Differences in pre-ejection period values between TGA patients with and without aortic valve regurgitation.**

| Aortic valve regurgitation | | No | Yes | P |
| --- | --- | --- | --- | --- |
| **PEP** | Sleep | 102.6 ± 10.7 | 104.7 ± 11.8 | 0.682 |
|  | Quiet sitting | 93.9 ± 7.6 | 103.8 ± 13 | 0.060 |
|  | Active sitting | 97.2 ± 11.7 | 104.9 ± 14 | 0.188 |
|  | Moderate PA | 80.2 ± 7.6 | 87.8 ± 11.6 | 0.105 |
|  | Heavy PA | 78.4 [78.1-81] | 75.6 ± 12.8 | 0.146 |

Data expressed as media ± SD or median [IQR]

*PEP*: pre-ejection period

**Supplementary Table S4. Correlation analysis between the left ventricle systolic function and the autonomic nervous system parameters.**

|  | **Activity level** | **TGA patients (n=26)** | **Healthy subjects (n=52)** |
| --- | --- | --- | --- |
| **HR vs 4ch LS** | Sleep | R=-0.374; p=0.065 | R=-0.015; p=0.917 |
|  | Quiet sitting | R=-0.380; p=0.056 | R=-0.009; p=0.949 |
|  | Active sitting | R=-0.390; p=0.049 | R=-0.008; p=0.954 |
|  | Moderate PA | **R=-0.209; p=-0.049** | R=0.015; p=0.919 |
|  | Heavy PA | R=-0.319; p=0.213 | R=-0.099; p=0.522 |
| **PEP vs 4ch LS** | Sleep | R=-0.382; p=0.072 | R=-0.167; p=0.248 |
|  | Quiet sitting | R=-0.351; p=0.085 | R=-0.271; p=0.066 |
|  | Active sitting | R=-0.220; p=0.291 | R=-0.52; p=0.722 |
|  | Moderate PA | R=0.010; p=0.962 | R=-0.093; p=0.525 |
|  | Heavy PA | R=0.095; P=0.726 | R=0.003; p=0.984 |
| **RSA vs 4ch LS** | Sleep | R=0.122; p=0.560 | R=0.114; p=0.429 |
|  | Quiet sitting | **R=0.437; p=0.026** | R=0.159; p=0.280 |
|  | Active sitting | **R=0.547; p=0.004** | R=0.264; p=0.064 |
|  | Moderate PA | R=0.301; p=0.136 | R=-0.010; p=0.944 |
|  | Heavy PA | **R=0.499; p=0.042** | R=0.181; p=0.240 |
| **RMSSD vs 4ch LS** | Sleep | R=0.241; p=0.246 | R=0.199; p=0.166 |
|  | Quiet sitting | **R=0.420; p=0.246** | R=0.127; p=0.390 |
|  | Active sitting | **R=0.491; p=0.011** | R=0.244; p=0.087 |
|  | Moderate PA | R=0.280, p=0.166 | R=-0.047; p=0.750 |
|  | Heavy PA | **R=0.488; p=0.047** | R=0.109; p=0.479 |
| **cRMSSD vs 4ch LS** | Sleep | R=0.193; p=0.356 | R=0.202; p=0.159 |
|  | Quiet sitting | **R=0.412; p=0.037** | R=0.153; p=0.300 |
|  | Active sitting | **R=0.477; p=0.014** | R=0.247; p=0.083 |
|  | Moderate PA | R=0.315; p=0.117 | R=-0.05; p=0.734 |
|  | Heavy PA | R=0.476; p=0.054 | R=0.130; p=0.402 |

Pearson or Spearman correlation when appropriate.

*HR*: heart rate; *PEP*: pre-ejection period; *RMSSD*: root of the mean square of successive normal-to-normal interval differences; *cRMSSD*: root of the mean square of successive normal-to-normal interval differences corrected by interbeat interval; *RSA*: respiratory sinus arrythmia; *4ch LS*: 4-chambers longitudinal strain.

**Supplementary Table S5. Correlation analysis between the cardiopulmonary exercise test parameters and the pre-ejection period**

|  | PEP  Sleep | PEP  Quiet sitting | PEP  Active sitting | PEP  Moderate PA | PEP  Heavy PA |
| --- | --- | --- | --- | --- | --- |
| WRpeak | **R=0.578 p=0.005** | **R=0.591 p=0.002** | **R=0.496 p=0.014** | **R=0.608**  **p=0.002** | R=0.344  p=0.211 |
| %WRpeak | R=0.108 p=0.634 | R=0.167 p=0.436 | R=0.377 p=0.070 | **R=0.410**  **p=0.047** | R=0.024  p= 0.931 |
| HRpeak | R=0.355 p=0.0105 | R=0.203 p=0.342 | R=0.114 p=0.597 | R= 0.145  p=0.499 | R=0.046  p=0.872 |
| %HRpeak | **R=0.454 p=0.034** | R=0.322 p=0.125 | R=0.113 p=0.535 | R=0.179  p= 0.402 | R=0.177  p= 0.529 |
| O_2_pulse | R=0.340 p=0.121 | **R=0.503 p=0.012** | **R=0.463 p=0.023** | **R=0.443**  **p=0.030** | R=0.278  p=0.316 |
| %O_2_ pulse | R=0.054 p=0.812 | R=0.371 p=0.075 | **R=0.449 p=0.028** | R=0.391  p=0.059 | R=-0.102  p=0.718 |
| VO_2_ peak | R=0.396 p=0.068 | **R=0.511 p=0.011** | **R=0.444 p=0.030** | **R=0.511**  **p= 0.011** | R=0.344  p=0.209 |
| %pred | R=-0.088 p=0.690 | R=-0.250 p=0.229 | R=-0.158 p=0.449 | R=0.351  p=0.085 | R=-0.366  p=0.163 |
| OUES | R=0.355 p=0.105 | **R=0.418; p=0.042** | **R=0.417 p=0.043** | R=0.335  p=0.110 | R=0.207  p=0.459 |
| % OUES | R=-0.189 p=0.400 | R=-0.096 p=0.656 | R=0.163 p=0.446 | R=-0.490  p=0.820 | R=-0.229  p=0.412 |
| VE | R=-0.397 p=0.068 | R=-0.145 p=0.498 | R=-0.203 p=0.341 | R=0.031  p=0.884 | R=0.015  p=0.959 |

Pearson or Spearman correlation when appropriate.

*HR_peak_*: maximal heart rate at peak exercise; *HR_peak_%*: % of the predicted values of HR_peak_; *IVS*: interventricular septum; *OUES*: oxygen uptake efficiency slope; *OUES*%: % of the predicted values of OUES; *PA*: physical activity; *PEP*: pre-ejection period; *RER_peak_*: respiratory exchange ratio at peak exercise; *RV*: right ventricle; *VCO_2_*: carbon dioxide production; *VE*: minute ventilation; *VO_2peak_*: oxygen uptake at peak exercise; *VO_2peak_%*: % of the predicted values of VO_2peak_; *VSD*: ventricular septal defect; *WR_peak_*: peak work rate; *WR_peak_%*: % of the predicted values of WR_peak_.

**Supplementary Table S6. Correlation analysis between the metabolic equivalent task score and the autonomic nervous system parameters.**

|  | **Activity level** | **TGA patients (n=26)** | **Healthy subjects (n=52)** |
| --- | --- | --- | --- |
| **HR vs METs** | Sleep | R=-0.113; p=0.627 | R=-0.080; p=0.584 |
|  | Quiet sitting | R=-0.307; p=0.164 | R=-0.274; p=0.062 |
|  | Active sitting | R=-0.219; p=0.328 | R=-0.056; p=0.702 |
|  | Moderate PA | R=-0.098; p=0.664 | R=-0.161; p=0.274 |
|  | Heavy PA | R=-0.301; p=0.317 | R=-0.161; p=0.307 |
| **PEP vs METs** | Sleep | R=-0.048; p=0.841 | R=0.098; p=0.504 |
|  | Quiet sitting | R=-0.370; p=0.090 | R=0.187; p=0.212 |
|  | Active sitting | R=-0.112; p=0.620 | R=-0.055; p=0.710 |
|  | Moderate PA | R=0.015; p=0.946 | R=0.019; p=0.897 |
|  | Heavy PA | R=0.137; p=0.655 | R=0.089; p=0.575 |
| **RSA vs METs** | Sleep | R=-104; p=0.654 | R=-0.197; p=0.175 |
|  | Quiet sitting | R=-0.215; p=0.336 | R=-0.062; p=0.678 |
|  | Active sitting | R=-0.331; p=0.133 | R=-0.255; p=0.078 |
|  | Moderate PA | R=-0.014; p=0.950 | R=0031.; p=0.833 |
|  | Heavy PA | R=0.164; p=0.593 | R=-0.021; p=0.895 |
| **RMSSD vs METs** | Sleep | R=-0.030; p=0.897 | R=-0.026; p=0.857 |
|  | Quiet sitting | R=-0.125; p=0.579 | R=0.069; p=0.360 |
|  | Active sitting | R=-0.110; p=0.626 | R=-0.134; p=0.645 |
|  | Moderate PA | R=0.010; p=0.966 | R=0.169; p=0.251 |
|  | Heavy PA | R=0.137; p=0.655 | R=0.036; p=0821 |
| **cRMSSD vs METs** | Sleep | R=-0.052; p=0.821 | R=-0.051; p=0.726 |
|  | Quiet sitting | R=-0.194; p=0.388 | R=0.038; p=0.800 |
|  | Active sitting | R=-0.150; p=0.506 | R=-0.142; p=0.332 |
|  | Moderate PA | R=-0.050; p=0.824 | R=0.134; p=0.365 |
|  | Heavy PA | R=0.170; p=0.578 | R=0.001; p=0.996 |

Pearson or Spearman correlation when appropriate.

*HR*: heart rate; *METs*: metabolic equivalent task score; *PEP*: pre-ejection period; *RMSSD*: root of the mean square of successive normal-to-normal interval differences; *cRMSSD*: root of the mean square of successive normal-to-normal interval differences corrected by interbeat interval; *RSA*: respiratory sinus arrythmia
